# Supplementary material for: Role of PKN1 in Retinal Cell Type Formation
Source: Int J Mol Sci. 2024 Feb 29;25(5):2848. doi: 10.3390/ijms25052848 (PMC10931565; doi:10.3390/ijms25052848)
Supplement: Supplementary file 1 [file ijms-25-02848-s001.zip › ijms-2864320-supplementary.pdf]

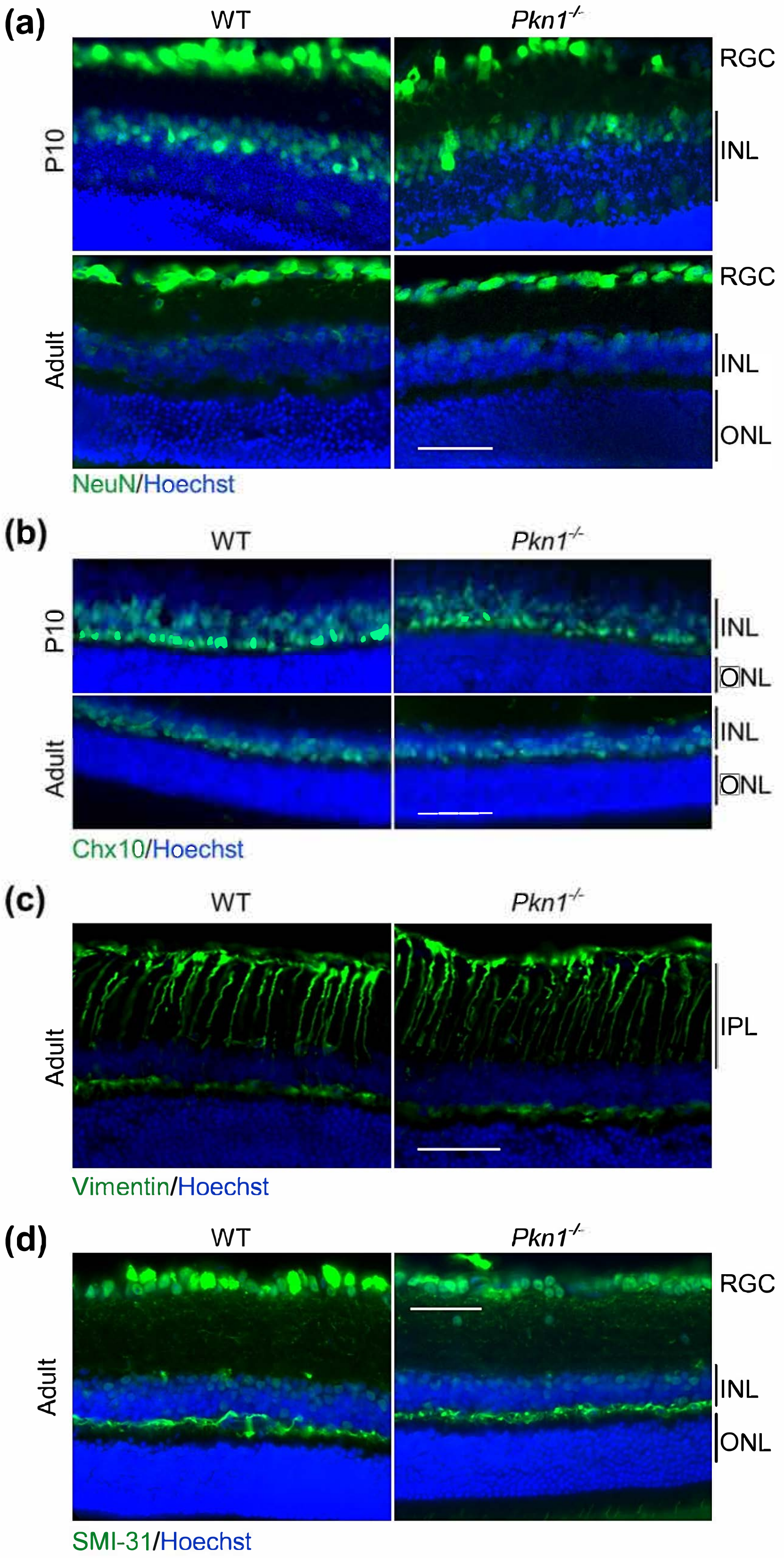

Supplemental Figure S1: Staining of retinal sections for various neuronal mark-ers. (**a**) Retinal sections from P10 old and adult animals were stained for NeuN and Hoechst (see Table 1 and Table 2 in the main manuscript for analysis and n-numbers). (**b**) Retinal sections from P10 old and adult animals were stained for Chx10 and Hoechst (see Table 1 in the main manuscript for analysis and n-numbers). (**c**) Adult retinal sections were stained for vimentin and Hoechst to visualize Müller glia cells. Images are representative of 3-4 separate animals per genotype. (**d**) Adult retinal sections were stained for SMI-31 and Hoechst (see Table 2 in the main manuscript for analysis and n-numbers). INL: inner nuclear layer; IPL: inner plexiform layer; ONL: outer nuclear layer; RGC: retinal ganglion cell layer. All scale bars refer to 50 µm.
